# Addressing Class Imbalance in Fetal Health Classification: Rigorous Benchmarking of Multi-Class Resampling Methods on Cardiotocography Data

**DOI:** 10.3390/diagnostics16030485

**Published:** 2026-02-05

**Authors:** Zainab Subhi Mahmood Hawrami, Mehmet Ali Cengiz, Emre Dünder

**Affiliations:** 1 Ministry of Higher Education and Scientific Research—KRG, Kirkuk Main Road, Erbil 44001, Kurdistan Region, Iraq; zaianbsubhi@gmail.com; 2 Department of Mathematics and Statistics, College of Science, Imam Mohammad Ibn Saud Islamic University (IMSIU), Riyadh 11432, Saudi Arabia; 3 Department of Statistics, Faculty of Science, Ondokuz Mayıs University, Samsun 55139, Turkey

**Keywords:** cardiotocography, fetal health classification, machine learning, class-imbalanced data

## Abstract

**Background/Objectives**: Fetal health is essential in prenatal care, influencing both maternal and fetal outcomes. Cardiotocography (CTG) monitors uterine contractions and fetal heart rate, yet manual interpretation exhibits significant inter-examiner variability. Machine learning offers automated alternatives; however, class imbalance in CTG datasets where pathological cases constitute less than 10% leads to poor detection of minority classes. This study aims to provide the first systematic benchmark comparing five resampling strategies across seven classifier families for multi-class CTG classification, evaluated using imbalance-aware metrics rather than overall accuracy alone. **Methods**: Seven machine learning models were employed: Naïve Bayes (NB), Random Forest (RF), Linear Discriminant Analysis (LDA), k-Nearest Neighbors (KNN), Linear Support Vector Machine (SVM), Multinomial Logistic Regression (MLR), and Multi-Layer Perceptron (MLP). To address class imbalance, we evaluated the original unbalanced dataset (base) and five resampling methods: SMOTE, BSMOTE, ADASYN, NearMiss, and SCUT. Performance was evaluated on a held-out test set using Balanced Accuracy (BACC), Macro-F1, the Macro-Matthews Correlation Coefficient (Macro-MCC), and Macro-Averaged ROC-AUC. We also report per-class ROC curves. **Results**: Among all models, RF proved most reliable. Training on the original distribution (base) yielded the highest BACC (0.9118), whereas RF combined with BSMOTE provided the strongest class-balanced performance (Macro-MCC = 0.8533, Macro-F1 = 0.9073) with a near-perfect ROC-AUC (approximately 0.986–0.989). Overall, resampling effects proved model dependent. While some classifiers achieved optimal performance on the natural class distribution, oversampling techniques, particularly SMOTE and BSMOTE, demonstrated significant improvements in minority class discrimination and class-balanced metrics across multiple model families. Notably, certain models benefited substantially from resampling, exhibiting enhanced Macro-F1, BACC, and minority class recall without sacrificing overall accuracy. **Conclusions**: These findings establish robust, model-agnostic baselines for CTG-based fetal health screening. They highlight that strategic oversampling can translate improved minority class discrimination into clinically meaningful performance gains, supporting deployment in cost-sensitive and threshold-aware clinical settings.

## 1. Introduction

Reducing fetal mortality and maintaining continuous surveillance of fetal health status are essential for ensuring the well-being of both the mother and the fetus [1]. Therefore, fetal heart rate (FHR) monitoring through cardiotocography (CTG) is a widely adopted technology for assessing fetal well-being during pregnancy and labor [2]. From a clinical perspective, CTG monitoring plays a significant role in determining obstetric outcomes. During pregnancy, women undergo substantial physiological and morphological changes that may influence fetal development and movement. Recognizing these changes is essential for healthcare providers to distinguish between normal physiological adaptations and potential pathological conditions that could compromise maternal and fetal health. Given these considerations, effective fetal monitoring is crucial in reducing adverse perinatal outcomes. Studies suggest that increased fetal movements may have a protective effect against cesarean delivery [3]. Healthcare professionals recommend routine assessments to evaluate fetal health, with CTG being widely acknowledged as a standard method for monitoring both fetal and maternal conditions during pregnancy [4]. Despite its widespread use, CTG interpretation frequently depends on the clinician’s expertise, which introduces variability in assessments and increases the likelihood of misinterpretation. Conventional CTG analysis may generate false alarms or fail to detect genuine signs of fetal distress, leading to either unnecessary interventions or insufficient monitoring.

The consequences of such misinterpretation extend beyond immediate clinical decisions. The burden of preventable perinatal morbidity extends beyond immediate clinical outcomes. Hypoxic-ischemic encephalopathy (HIE), a severe consequence of perinatal asphyxia, has an incidence of approximately 1.5 per 1000 live births in high-income countries. In low- and middle-income countries, estimates range from approximately 2.3 to 26 per 1000 live births, depending on region, diagnostic definitions, and access to care [5]. These statistics underscore the critical importance of effective fetal surveillance strategies, particularly in detecting and preventing hypoxic injury during labor and delivery.

On a global scale, the worldwide prevalence of adverse perinatal outcomes highlights the necessity of enhancing fetal surveillance systems. According to the United Nations Interagency Group for Child Mortality Estimation (UN IGME) report, approximately 2 million stillbirths occur annually, as of 2023, with 46% happening during labor. The majority of intrapartum deaths (98%) occur in low- and middle-income countries, where high rates of home births and unattended deliveries remain significant challenges. Improving access to care, particularly in these settings, is critical to reducing intrapartum-related deaths and disabilities.

Central to effective fetal surveillance is the accurate interpretation of CTG recordings. CTG analysis involves evaluating five key features: baseline FHR (typically 110–160 beats per minute), baseline variability (fluctuations in the baseline rate), accelerations (transient increases in FHR), decelerations (transient decreases), and uterine contraction patterns. The International Federation of Gynecology and Obstetrics (FIGO) updated guidelines in 2015 established a three-tier classification system. This system categorizes CTG traces as normal, suspicious, or pathological based on combinations of these features [6]. One of the major challenges in intrapartum fetal monitoring is that, despite the international classification systems for CTG patterns, there is a very high false-positive rate of around 60% [7]. The diagnostic accuracy of admission CTG differs across studies. Positive predictive values have been reported between 19% and 88%, while negative predictive values range from 88.6% to 100% [8,9,10,11,12].

Adding to these diagnostic challenges, continuous CTG monitoring produces substantial volumes of data, requiring considerable time for healthcare providers to analyze and interpret accurately [13]. As a result, computerized CTG analysis has been seen as a promising solution to overcome these issues [14]. Moreover, automated early warning systems that integrate computerized cardiotocography with perinatal health parameter databases can provide objective alerts for patients at risk, potentially improving outcomes [15]. To address these limitations, advancements in computerized fetal heart rate monitoring, application of big data and artificial intelligence, and innovations in home and remote monitoring are being explored [16].

To address these limitations in manual CTG interpretation, researchers have increasingly turned to machine learning approaches. The integration of machine learning into CTG analysis has gained momentum over the past decade. Studies on fetal health classification focus on the analysis of cardiotocographic data used in the evaluation of fetal health. Mehbodniya et al. [1] investigated the prediction of fetal health status from CTG data using various ML algorithms, including SVM, RF, MLP, and KNN. In this study, the authors classified fetal health status as either normal, suspect, or pathological. The RF algorithm and XGBoost exhibited the best performance, demonstrating higher accuracy, sensitivity, and F1-score values. Subsequently, Ocak [17] applied genetic algorithm-based feature selection with SVM classifiers, achieving 99.3% and 100% accuracy for normal and pathological CTG classification, respectively. The proposed scheme outperformed ANN and ANFIS-based methods. Hoodbhoy et al. [18] compared ten different machine learning algorithms on CTG data. The study employed SMOTE to address class imbalance, and the classification model developed using the XGBoost technique had the highest prediction accuracy for an adverse fetal outcome.

While these studies demonstrate the potential of machine learning for CTG-based fetal health classification, a critical challenge that affects model reliability remains largely underexplored: the need for high-quality and balanced datasets. However, real-world medical datasets are frequently imbalanced due to naturally skewed outcome distributions. Many existing studies have overlooked this issue despite its critical influence on model performance and clinical validity. Class imbalance biases models toward majority classes, reducing their capacity to identify minority patterns [19]. This asymmetry in clinical consequences highlights the importance of appropriate evaluation metrics. Standard accuracy measures can be misleading under severe class distribution skews, often favoring the majority class while masking poor performance on minority classes [20]. Models trained on balanced data typically achieve more stable convergence, enhanced interpretability, and improved detection of minority outcomes. When natural class balance is not achievable, techniques such as resampling, class weighting, or data augmentation help mitigate bias and enhance both fairness and clinical utility.

To address these challenges, this study investigates multi-class fetal health classification from CTG data under conditions of class imbalance. We evaluate seven machine learning algorithms: Naïve Bayes (NB), Random Forest (RF), Linear Discriminant Analysis (LDA), k-Nearest Neighbors (KNN), Linear Support Vector Machine (SVM), Multinomial Logistic Regression (MLR), and Multi-Layer Perceptron (MLP).

The selection of these seven algorithms was motivated by their representation of fundamentally different learning paradigms: Naïve Bayes represents probabilistic classifiers that assume feature independence, while Random Forest exemplifies ensemble methods that aggregate multiple decision trees to reduce variance. Linear Discriminant Analysis and Support Vector Machines represent linear approaches that seek optimal separating boundaries, whereas k-Nearest Neighbors relies on local instance similarity without explicit model construction. Multinomial Logistic Regression extends classical regression to multi-class settings, and Multi-Layer Perceptron captures non-linear relationships through layered neural architectures. As noted by Fernández et al. [21], different classifier families exhibit varying sensitivity to class imbalance, making algorithmic diversity essential for comprehensive evaluation.

To mitigate the effects of class imbalance, each algorithm was tested across five resampling strategies: SMOTE (Synthetic Minority Oversampling Technique), Borderline-SMOTE (BSMOTE), ADASYN (Adaptive Synthetic Sampling), NearMiss, and SCUT. These five resampling techniques were selected to represent distinct balancing strategies. SMOTE and its variants (Borderline-SMOTE, ADASYN) generate synthetic minority samples through interpolation, with each variant addressing specific limitations of the original algorithm. NearMiss applies undersampling by removing majority class instances based on their distance to minority samples, while SCUT combines both oversampling and undersampling in a hybrid framework. Comparing these approaches allows us to determine whether synthetic data generation or majority class reduction is more effective for CTG classification.

We also included the raw, unbalanced dataset as a baseline. Model performance is assessed on a held-out test set using four complementary metrics: Balanced Accuracy (BACC), Macro-F1-Score, Macro-Matthews Correlation Coefficient (Macro-MCC), and Macro-Averaged ROC-AUC. Per-class ROC curves are also presented to illustrate the discriminative performance for each fetal health category.

The primary contributions of this study are threefold: (1) systematic comparison of five resampling strategies across seven classifiers, addressing the fact that most studies evaluate only one or two methods; (2) rigorous evaluation using four imbalance-aware metrics rather than overall accuracy alone; and (3) practical guidelines for selecting classifier–resampling combinations suitable for clinical deployment. To the best of our knowledge, this is the first benchmarking study to systematically compare multiple resampling strategies across diverse classifier families specifically for imbalanced multi-class fetal health classification using class-balanced evaluation metrics.

The remainder of this paper is organized as follows: Section 2 presents related works, summarizing previous studies on fetal health classification using CTG data and identifying the current gaps motivating this research. Section 3 details the proposed methodology, including the dataset description, workflow design, algorithmic structure, and imbalance handling strategies. Section 3.1 outlines the machine learning algorithms and resampling techniques employed in model development. Section 3.2 describes the model evaluation framework, including the performance metrics adapted for multi-class and imbalanced data. Section 4 presents the results and discussion that interpret the experimental results, including ROC analyses. Section 5 concludes with key findings and outlines directions for future research.

## 2. Related Work

For more than three decades, CTG’s predictive capacity has remained controversial despite its widespread use in fetal risk assessment. Reported sensitivities range from 2% to 100%, while specificities vary between 37% and 100% [6,22]. This wide variability is driven largely by subjective visual interpretation. This motivates the adoption of ML to standardize analysis and improve discrimination among normal, suspect, and pathological fetal states. Using the canonical UCI CTG dataset (2126 records, 21 features, 3 class labels), multiple studies have demonstrated that classical ML methods can achieve high performance. Methods such as RF, SVM, Decision Tree (DT), and KNN have exceeded 90% accuracy in various evaluations [23,24].

Early studies confirmed this promise but also exposed limitations. Sahin et al. [25] compared eight algorithms including Logistic Regression, RF, ANN, KNN, DT, SVM, RBFN, and CART using ten-fold cross-validation. They found RF to be the most reliable for binary classification (normal vs. pathological). Spilka et al. [26] modeled intrapartum FHR trajectories with sparse SVMs, achieving approximately 70% accuracy. This underscored the challenge of noisy, time-varying signals. Complementary studies reported additional findings. Ramla et al. [27] achieved approximately 88.9% accuracy using DT techniques. Madiraju et al. [28] showed that MLPs can learn non-linear relations within FHR variability, accelerations, and decelerations. Collectively, these works established feasibility while highlighting the need to manage class imbalance and improve generalization.

Subsequent research incorporated feature selection, optimization, and cost-sensitive methods to strengthen performance. Piri et al. [29] benchmarked an association-based classifier against DT, Linear Regression, SVM, KNN, GNB, RF, and XGBoost. They observed modest gains after feature selection (83% to 84% accuracy). The authors noted that insufficient data augmentation can promote overfitting and hinder generalization. Vani et al. [30] introduced a weighted DNN and evaluated its performance using sensitivity, specificity, F1-score, and G-mean. The weighted DNN achieved a G-mean of 91% for positive-class detection, outperforming the SVM baseline. However, limited hyperparameter tuning likely constrained overall performance. Building on this work, Piri et al. [31] employed an evolutionary multi-objective genetic algorithm (MOGA-CD) to identify influential predictors in the CTG dataset. Using the selected features, DT, SVM, GNB, RF, and XGBoost achieved the highest classification accuracies among the evaluated models. Related contributions emphasized interpretability and error reduction. Zhou et al. [32] highlighted the value of DTs for identifying high-risk cases. Li et al. [33] showed that ML can reduce subjective error in CTG interpretation. Pradhan et al. [34] reported that RF achieved approximately 99% accuracy when compared with Logistic Regression, KNN, and Gradient-Boosting Machines.

As pipelines matured, explicit imbalance handling and ensemble methods became decisive. Rahmayanti et al. [35] compared ANN, LSTM, XGBoost, SVM, KNN, LightGBM, and RF across three scenarios. These scenarios varied outlier control, VIF-based collinearity filtering, and upsampling with SMOTE. LightGBM was uniquely consistent, achieving near-perfect performance (accuracy, F1-score, and AUC approximately 0.99) across settings. Kaliappan et al. [36] proposed an ensemble voting scheme combining KNN, GNB, and AdaBoost with Monte Carlo cross-validation. They reported approximately 99% accuracy, high recall (approximately 98%), and an F1-score of approximately 97%. Regmi and Shah [37] evaluated dimensionality-reduction pipelines (PCA, LDA) alongside attention-based and tabular learners. Their TabNet model achieved 94.36% accuracy on the fetal health dataset. Within classical pipelines, the LDA-based combination outperformed the RF with PCA variant. Their best pipeline for prenatal abnormality classification reached 91.13% accuracy. Two studies by Salini et al. [38] applied LR, RF, DT, SVM, KNN, GBM, and voting models. They repeatedly found RF to be strongest, with approximately 93% accuracy. Addressing imbalance from a different angle, Zeng et al. [39] proposed an ensemble cost-sensitive SVM (ESVM) using time–frequency features to distinguish normal versus abnormal fetuses.

Very recent benchmarks reinforce these patterns while extending to meta-learning and broader deep learning comparisons. Nazlı et al. [40] evaluated a large panel of algorithms: CatBoost, DT, ExtraTrees, GB, KNN, LightGBM, RF, SVM, ANN, and DNN with SMOTE to correct imbalance. They found LightGBM best (accuracy = 90.73%, balanced accuracy = 91.34%), confirming its robustness on three-class CTG. Ahmed et al. [13] combined seven ML and five DL models under SMOTE. A stacking meta-classifier surpassed all individual learners (accuracy = 98.9%, precision = 99.0%, recall = 98.6%, F1 = 99.3%, AUC = 99.8%). This suggests practical suitability for real-time CTG decision support. Bhukya et al. [41] compared wide-ranging ML methods (KNN, DT, SVM, RF, SGD, GB, AdaBoost, XGBoost) and DL methods (CNN, LSTM, BiLSTM). They found RF to be highest (approximately 99.39%), with XGBoost and SVM close behind. Among DL methods, BiLSTM and LSTM outperformed CNN but were slightly less stable on the suspect class.

Most prior studies report overall accuracy, which can be misleading for imbalanced datasets by favoring majority class performance. While some studies achieve higher accuracy values, these metrics may not reflect true performance on minority classes such as pathological cases. In contrast, our study employs class imbalance-robust metrics (BACC, Macro-F1, Macro-MCC, and Macro-Averaged ROC-AUC) that provide fairer evaluation across all fetal health categories. Unlike previous studies that addressed class imbalance through isolated techniques or focused on binary classification scenarios, we comprehensively benchmark five resampling strategies (SMOTE, BSMOTE, ADASYN, NearMiss, SCUT) across seven diverse model families (NB, RF, LDA, KNN, Linear SVM, MLR, and MLP). Our methodological rigor distinguishes this work through several key practices. A detailed methodology is presented in Section 3.

## 3. Methodology

### 3.1. Dataset Overview

CTG provides valuable information on FHR and UC, which are crucial to assessing the well-being of the fetus. Historically, CTG traces have been printed and interpreted manually, a process that is time-consuming and susceptible to inter-observer variability. Automating classification can provide timely decision support, facilitating earlier diagnosis and more efficient use of clinical resources.

To enable such automation, a standardized classification framework is essential. The CTG classification system categorizes fetal heart rate recordings into three distinct patterns, each carrying specific clinical implications [42,43]. These categories are as follows: normal, pathological, and suspect.

In a normal tracing, the baseline fetal heart rate ranges between 110 and 160 beats per minute, with variability maintained at 5 to 25 beats per minute and no repetitive decelerations present. These parameters indicate that fetal heart rate and uterine contraction patterns fall within healthy physiological boundaries, with no immediate concerns regarding fetal well-being. Clinically, such findings suggest adequate fetal oxygenation and typically require only routine monitoring without any intervention.

A pathological pattern, by contrast, is characterized by a baseline heart rate falling below 100 beats per minute, along with variability that may be reduced, elevated, or sinusoidal in nature. This category also includes recurrent late or prolonged decelerations lasting beyond 30 min, or beyond 20 min if variability is already reduced, as well as any single deceleration extending past 5 min. These abnormalities point to a high probability of fetal hypoxia or acidosis and demand a prompt clinical response. Management may involve conservative approaches such as maternal repositioning and oxygen supplementation, or, in more urgent situations, expedited delivery.

The suspect category occupies the middle ground between these two extremes. It applies when one or more normal criteria are not met yet no clearly pathological features are observed. This pattern reflects irregularities in the fetal heart rate that, while not immediately alarming, warrant closer attention and may signal developing problems. Clinically, suspect tracings call for heightened vigilance, more frequent monitoring, and preparedness to intervene should the pattern worsen.

Given these clinical consequences, accurate identification of pathological cases is particularly critical. To develop and evaluate automated classification models based on this framework, a reliable dataset with expert-validated labels is required. This study used the publicly available Cardiotocography (CTG) dataset from the University of California Irvine Machine Learning Repository (UCI ML Repository) [44]. The fetal CTG records were generated by SisPorto 2.0 (Speculum, Lisbon, Portugal), a software system for automated CTG analysis [45]. SisPorto 2.0 computes groups of 194 indicators commonly used in fetal monitoring, including fetal heart rate (FHR) parameters, uterine contractions (UC), fetal movements, short- and long-term variability measures, and histogram-based features aligning with standard clinical practice. These variables form the feature set used for model development in this work.

**Figure 1:**
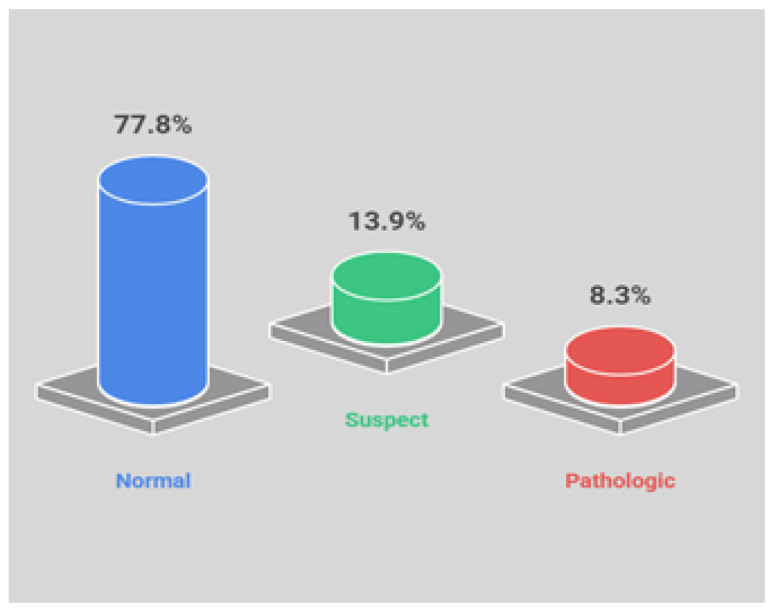
Percentage distribution of fetal health classes (normal, suspect, pathological).

The dataset is a widely used benchmark in fetal health classification because of its structured feature set, expert annotations, and clear class labels. It comprises 2126 third-trimester cases, each with 21 attributes describing fetal status. Three specialist obstetricians independently evaluated each CTG and assigned a consensus label, which serves as the gold standard. Fetal status is categorized into three classes: normal (1655 records; 77.8%), suspect (295; 13.9%), and pathological (176; 8.3%), as illustrated in Figure 1. The variables used to quantify FHR and UC, along with histogram and variability features, are summarized in Table 1 [46].

As is evident from this distribution, the dataset exhibits considerable class imbalance, with pathological cases representing only 8.3% of all records. In this study, we address the class imbalance problem inherent in CTG-based fetal health data by evaluating multiple resampling strategies and their impact on machine learning model performance for multi-class fetal health classification [40].

### 3.2. Workflow of the Model

**Figure 2:**
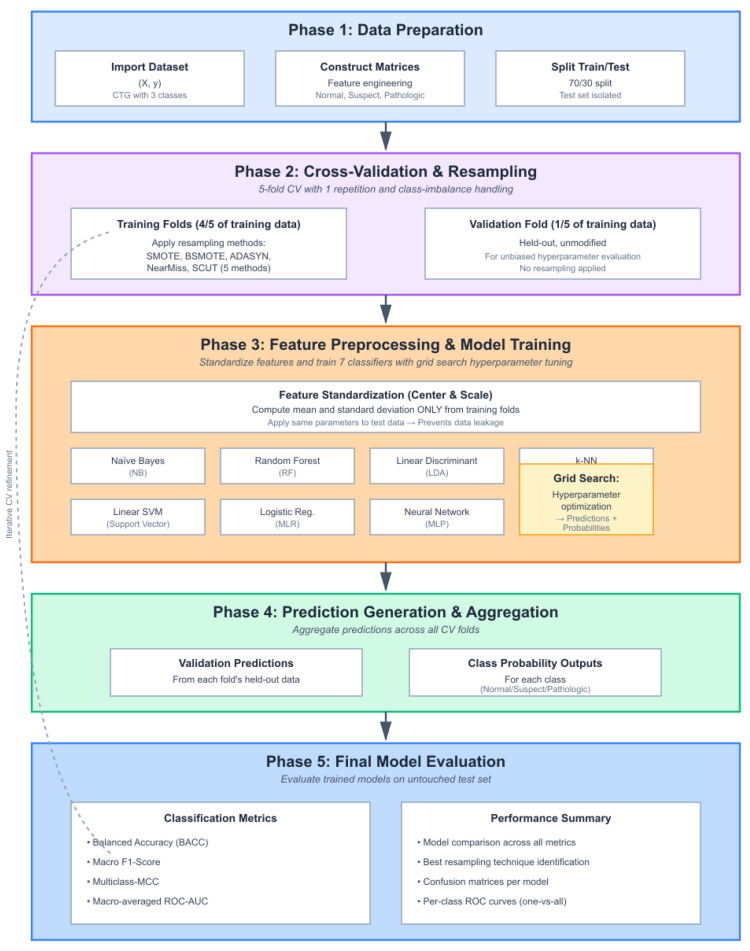
Workflow of the proposed CTG-based fetal health classification model.

To address the challenges of multi-class and imbalanced CTG data, we designed a structured machine learning pipeline. This pipeline integrates stratified data partitioning, cross-validation, preprocessing, class balancing, classifier training, and performance evaluation. The workflow was implemented to ensure robustness and reproducibility and prevent data leakage. We applied seven different classifiers and evaluated them using both threshold-based and ranking-based performance measures. The following steps outline the detailed workflow, from data preparation to classifier training and final evaluation. Figure 2 illustrates the complete process, highlighting the adaptability and effectiveness of our approach for fetal health classification.

Workflow Steps

1. Data acquisition and labeling: The CTG dataset is obtained with three outcome classes: normal, suspect, and pathologic.
2. Train–test split: The data is partitioned using a 70/30 stratified split. The test set is set aside as an independent validation cohort for unbiased final evaluation.
3. Cross-validation for hyperparameter tuning: Within the training set, 5-fold cross-validation is performed. During each fold, four parts are used for training and one part serves as the validation set. This process is used exclusively for selecting the best hyperparameters. A fixed random seed (seed = 123) is used to ensure identical cross-validation folds across all models, enabling a fair performance comparison.
4. Class imbalance handling: Five different resampling methods are applied: SMOTE, BSMOTE, ADASYN, NearMiss, and SCUT. Each method is applied exclusively to the training data to address class imbalance and prevent data leakage. The original unbalanced training set is also retained as a baseline.
5. Balanced training data: Resampling produces balanced training sets with corrected class proportions. This reduces majority class bias during model training.
6. Feature preprocessing: Both training and test sets are standardized. The mean and standard deviation are computed only on the training data and then these same parameters are applied to the test data.
7. Model training and hyperparameter tuning: Seven machine learning algorithms are trained: NB, RF, LDA, KNN, SVM, MLR, and MLP. Hyperparameters are tuned via grid search within the cross-validation process. Probability outputs are generated for ROC analysis.
8. Prediction generation: Each trained model is used to produce predictions and probabilities on the held-out validation fold, aggregating results across folds.
9. Final evaluation: The retrained models are evaluated on the untouched test set. Performance is measured using BACC, Macro-F1, Macro-MCC, and Macro-Averaged ROC-AUC. Per-class ROC curves are generated using a one-vs-all approach, and individual class AUC values are averaged to compute the Macro-AUC.

### 3.3. Algorithms

Machine learning (ML) is a branch of artificial intelligence that extracts patterns from raw data through algorithmic modeling. Recent advances have accelerated its use in healthcare, enabling high-performing models for classifying fetal health. These developments provide a foundation for future work, allowing researchers to build on prior successes and refine predictive tools. ML shows substantial promise for streamlining fetal health classification and supporting clinical decision-making. By understanding what drives model predictions, we can improve model design and ultimately enhance patient outcomes [13,47]. ML is now widely applied across perinatal research. Prior studies have used ML to estimate fetal weight [48], assess the probability of fetal hypoxia [49], and predict fetal growth and gestational age [50]. In this study, we focus specifically on classifying fetal health from cardiotocography (CTG) data using machine learning approaches [35].

We evaluate seven different algorithms, each tuned over relevant hyperparameters to ensure a fair comparison. Models are trained with 5-fold cross-validation and standardized features (centering and scaling). To test robustness under class imbalance, we assess performance on multiple resampled datasets. These include the raw data as well as SMOTE, BSMOTE, ADASYN, NearMiss, and SCUT variants. The following sections describe the algorithms, their key characteristics, and the hyperparameter settings used in this work.

#### 3.3.1. NB

This is a probabilistic classification method based on Bayes’ theorem. It estimates the probability of an event using prior information about related conditions [51]. The model assumes conditional independence among features within each class, even though some interdependence may exist in practice [52,53]. Despite its simplicity, NB is computationally efficient. It often performs well on text classification tasks, particularly when combined with TF-IDF features [54]. However, the independence assumption can limit its performance on more complex datasets [55]. In this study, we tuned NB using three key hyperparameters: First, usekernel enables or disables kernel density estimation for continuous predictors. Second, fL applies Laplace smoothing to address zero-frequency issues. Third, adjust controls the kernel bandwidth to adjust the smoothness of density estimation.

#### 3.3.2. RF

This is a supervised ensemble method that extends bootstrap aggregating (bagging). It constructs multiple decision trees and aggregates their predictions to improve accuracy and reduce overfitting. Each tree is trained on a bootstrap sample of the data. At each split, a random subset of features is selected to increase diversity among trees [56]. For classification tasks, the final output is determined by majority voting across all trees. As the number of trees grows, the generalization error stabilizes. This error depends on both the strength of individual trees and their correlation [57]. In this study, RF was tuned by varying the number of variables considered at each split (mtry). We tested several proportions of the total predictors: $\sqrt{p},\frac{3p}{4},\frac{p}{2},\frac{p}{4},\frac{p}{10}$, where *p* represents the number of features. The forest size was fixed at 1000 trees (ntree = 1000).

#### 3.3.3. KNN

This is one of the simplest and earliest classification algorithms [58]. It classifies a new observation based on the labels of its nearest neighbors in the training set. The method assumes that similar samples are likely to belong to the same class. The parameter k specifies the number of neighbors considered in the voting process. Different values of k can produce different classification results for the same input. KNN is a non-parametric supervised learning method that does not require distributional assumptions. This makes it straightforward to implement and widely applicable [59]. In this study, KNN was tuned by varying the number of neighbors (k). We tested k values of 1, 3, 5, 7, 9, and 11.

#### 3.3.4. Linear SVMs

Support Vector Machines classify data by finding a hyperplane that maximizes the margin between classes in a multi-dimensional feature space. Each observation is mapped to an n-dimensional space, and the algorithm identifies the hyperplane that best separates the classes. In this study, we used a linear SVM variant. The performance of the SVM largely depends on the regularization parameter, which controls the trade-off between margin maximization and classification errors. We tuned the cost parameter (C) at values of 0.1, 1, 10, and 100, with probability modeling enabled to generate class probabilities [53,60,61,62,63].

#### 3.3.5. LDA

Linear Discriminant Analysis is a supervised machine learning algorithm used for classification and dimensionality reduction. Introduced by R.A. Fisher in 1936, it has become a foundational method in statistical pattern recognition [64,65]. LDA identifies linear combinations of features that maximize class separation. It does this by minimizing within-class variance while maximizing between-class variance. This projects high-dimensional data into a lower-dimensional subspace that ensures maximum class separability [66,67]. By representing samples through these linear projections, LDA constructs base vectors that capture class differences, enabling both dimensionality reduction and effective classification [68]. In this study, LDA was implemented without additional hyperparameter tuning as it does not require a parameter grid (grid = NULL).

#### 3.3.6. MLR

Multinomial Logistic Regression is an extension of binary logistic regression designed for situations where the dependent variable has more than two nominal, unordered categories. It models the probability of each outcome category relative to a baseline using maximum-likelihood estimation [69,70]. This method is widely used for predicting outcomes with multiple classes, such as health status, job roles, or product preferences. Similar to binary logistic regression, MLR offers a flexible approach for modeling categorical outcomes [71,72]. In this study, MLR was tuned using a regularization parameter (decay) to prevent overfitting. We tested values of 0 and $1\times 10^{-10}$. The model was trained with a maximum weight capacity of 100,000 and tracing disabled to improve computational efficiency.

#### 3.3.7. MLP

Multi-Layer Perceptron is a feedforward artificial neural network widely used in supervised learning tasks such as classification, regression, and function approximation [13,73]. An MLP consists of multiple layers of interconnected neurons. The outputs of one layer serve as inputs to the next. Each neuron applies an activation function to its weighted inputs and transmits the result forward. This enables the network to capture complex patterns in the data [74,75]. The model is typically trained using the backpropagation algorithm. This algorithm iteratively updates connection weights by minimizing prediction error. Known for its flexibility and predictive accuracy, MLP has been successfully applied across diverse domains [76]. In this study, the MLP was tuned by varying two key hyperparameters: First, the number of hidden units (size) was tested at 3, 5, 7, 9, and 11. Second, weight decay (decay) was tested at values of 0, $1\times 10^{-5},\;1\times 10^{-4},\;1\times 10^{-3},\;1\times 10^{-2},\;\mathrm{and}\;1\times 10^{-1}$. The maximum number of iterations was set to 300.

### 3.4. Class Imbalance and Resampling Methods

Class imbalance significantly affects both model performance and clinical utility. It introduces bias toward the majority class and impairs the detection of rare but clinically critical minority cases [77]. When one class contains far more observations than others, overall model performance degrades [78]. In typical imbalanced datasets, most samples belong to the dominant class while only a small fraction represent the minority [79]. Models trained on such data tend to favor the majority class and often fail to recognize minority patterns [80]. As a result, minority class instances, often the most critical to identify, are frequently misclassified.

Imbalanced datasets pose four recurring challenges: bias, class overlap, high-dimensional feature spaces, and limited sample sizes [81]. These issues are especially pronounced in healthcare applications, such as diabetes diagnosis and skin lesion classification, as well as other safety-critical domains [82]. In medical diagnostics, the consequences can be severe. Models biased toward the majority class may miss rare diseases, leading to delayed or missed treatment and compromised patient care [83,84]. Addressing class imbalance is therefore fundamental to building reliable, fair, and clinically useful machine learning systems.

This concern extends directly to cardiotocography (CTG) analysis, where machine learning methods face severe class imbalance in clinically relevant outcomes. Standard algorithms trained on imbalanced data often achieve high overall accuracy yet fail to detect minority cases, a pattern observed across medicine and engineering [85].

In CTG analysis specifically, prior studies showed mixed results for resampling techniques. Almadi et al. [86] reported that applying SMOTE to a CTG dataset reduced accuracy despite strong baseline performance. Khan et al. [87] used random oversampling to mitigate imbalance. Nazli et al. [40] found that SMOTE improved Balanced Accuracy and reduced error rates across multiple models. Piri and Mohapatra [88] showed that seven resampling techniques substantially improved performance for RF, SVM, and DT on the UCI CTG dataset. These findings highlight that, while resampling can be beneficial, its effectiveness depends on both the dataset and the model, requiring careful design and validation.

Resampling-based approaches, such as Synthetic Minority Oversampling and random oversampling, offer practical ways to modify the training distribution and achieve more balanced class representation [89,90,91]. Their effectiveness depends on several factors, including imbalance ratio, dataset size, dimensionality, and class overlap. Selecting an appropriate strategy is crucial, as different techniques perform better under different conditions [92]. Moreover, hybrid methods that combine complementary strengths may further improve minority class recognition [93].

In this study, we applied five resampling techniques: SMOTE, BSMOTE, ADASYN, NearMiss, and SCUT. These methods were used to generate balanced training sets from the imbalanced CTG dataset. They aimed to improve minority class representation and enhance classifier robustness. Unless otherwise noted, neighborhood-based algorithms used k = 5, and oversampling targeted class parity (oversampling ratio = 1). This means minority classes were expanded to approximately match the majority class size.

#### 3.4.1. SMOTE

This is an oversampling method that increases minority class representation to approach parity with the majority class [94]. Rather than simply duplicating existing observations, SMOTE synthesizes new minority samples through a more sophisticated process. It selects a minority instance, identifies its k-nearest minority neighbors, and generates new points through linear interpolation along the segments connecting them [95,96]. This approach enriches the minority region of the feature space, reduces the overfitting risk associated with simple duplication, and can improve downstream classifier performance [97].

#### 3.4.2. BSMOTE

Borderline-SMOTE extends SMOTE by focusing on the most informative samples. Instead of oversampling all minority instances, BSMOTE generates synthetic samples only for minority instances located near the decision boundary between classes, where misclassification is most likely to occur [98]. By concentrating on these borderline cases, BSMOTE produces fewer but more strategically placed synthetic instances. This focused approach improves class separability and reduces overfitting compared to standard SMOTE [99,100].

#### 3.4.3. ADASYN

This method addresses class imbalance by estimating the local distribution of the minority class and applying a weighted distribution to minority instances according to their learning difficulty [101]. Using the nearest neighbor technique, it generates synthetic instances in proportion to each instance’s difficulty, thereby balancing the dataset and enhancing classifier performance. The method offers two main advantages: First, it reduces bias from imbalanced class distributions. Second, it shifts the decision boundary toward hard-to-learn samples, improving minority class classification [102]. ADASYN assigns different weights to minority samples and automatically determines how many synthetic samples each should produce to achieve class balance [103].

#### 3.4.4. NearMiss

This is a family of undersampling methods that address class imbalance by removing the majority class samples closest to minority instances. The algorithm first computes distances between majority and minority observations. It then focuses on majority points that lie near the minority set, thereby concentrating training data around the decision boundary [104,105]. In practice, the method works as follows: For each minority sample, it identifies its nearest majority neighbors. It retains the majority points most informative for class separation and discards the remaining majority samples to achieve a more balanced dataset. This targeted reduction of the majority class improves class separability and can enhance model generalization to unseen data [106,107,108].

#### 3.4.5. SCUT

This method combines undersampling and oversampling to address class imbalance in multi-class datasets. The dataset is first divided into n subsets ($D_{1},\ldots ,\;D_{n}$), one for each class. The overall mean count m, representing the average number of instances across classes, is calculated. For classes with fewer instances than the mean, oversampling is applied until the class size reaches m. The SMOTE algorithm is used to determine the appropriate sampling percentage. For classes with more instances than the mean, undersampling is carried out to reduce their size to m. In this case, the Expectation Maximization (EM) clustering technique [109] is employed to identify clusters within each class. Instances are then randomly selected from these clusters so that the total equals m. Instead of fixing the number of samples per cluster, the selection is distributed flexibly to achieve uniform representation. The selected instances are merged to form the final balanced class. Classes that already contain exactly m instances remain unchanged [110].

### 3.5. Model Evaluation

Each classifier was evaluated on the held-out test set using the confusion matrix and a set of performance metrics specifically designed for imbalanced, multi-class data. The confusion matrix provides a structured summary of classification outcomes. It captures the counts of correct and incorrect assignments through four key components: true positives (TP), true negatives (TN), false positives (FP), and false negatives (FN). From these basic components, we can define the total number of actual positives as P = TP + FN and the total number of actual negatives as N = TN + FP [111].

Classification metrics are derived directly from the confusion matrix and do not depend on probability estimates. Instead, they provide straightforward and interpretable evaluations of a model’s ability to correctly categorize instances. In this study, we focused on four key metrics: BACC, Macro-F1-Score, Macro-MCC, and Macro-Averaged ROC-AUC. These metrics were specifically chosen to address the challenges of imbalanced multi-class classification.

- BACC: This metric mitigates the effect of class imbalance by averaging sensitivity (TP/P) and specificity (TN/N).
  (1) $$\mathrm{BACC}=\frac{1}{2}\left(\frac{TP}{P}+\frac{TN}{N}\right)$$
- Macro-F1-Score: The F1-score is the harmonic mean of precision and recall, providing a balanced measure that accounts for both false positives and false negatives. In multi-class settings, the Macro-F1 is computed by calculating the F1-score separately for each class and then averaging the results. For each class *i*, precision ($P_{i}$), recall ($R_{i}$), and the corresponding F1-score ($F1_{i}$) These are computed as follows:
  $$P_{i}=\frac{TP_{i}}{TP_{i}+FP_{i}},\;R_{i}=\frac{TP_{i}}{TP_{i}+FN_{i}},\;F1_{i}=\frac{2P_{i}R_{i}}{P_{i}+R_{i}}$$
  Finally, the Macro-F1-Score is obtained by taking the arithmetic mean across all *k* classes:
  (2) $$\mathrm{Macro}\text{-}F_{1}\;=\;\frac{1}{k}\sum_{i=1}^{k}F1_{i}$$
  This macro-averaging approach treats all classes equally, regardless of their size. This makes it particularly suitable for imbalanced datasets where minority class performance is critical. The Macro-F1 is widely applied across binary, multi-class, and multi-label classification problems, particularly in domains where class imbalance is prevalent [112,113].
- Macro-MCC: The Matthews Correlation Coefficient (MCC) evaluates the quality of binary classifications by incorporating all four entries of the confusion matrix. It ranges from +1 (perfect agreement) to −1 (complete disagreement), with 0 representing random prediction [114]. For multi-class problems, we employ a macro-averaging approach. MCC is computed separately for each class using a one-vs-rest strategy, where each class is treated as positive while all others are grouped as negative. The per-class MCC values are then averaged to obtain the Macro-Averaged MCC:
  (3) $$\mathrm{Macro}\text{-}\mathrm{MCC}\;=\;\frac{1}{k}\sum_{i=1}^{k}\frac{TP_{i}\;TN_{i}\;-\;FP_{i}\;FN_{i}}{\sqrt{\left(TP_{i}+FP_{i}\right)\left(TP_{i}+FN_{i}\right)\left(TN_{i}+FP_{i}\right)\left(TN_{i}+FN_{i}\right)}}$$
  where *k* represents the number of classes, and $TP_{i}$, $TN_{i}$, $FP_{i}$, and $FN_{i}$ denote the true positives, true negatives, false positives, and false negatives for class *i*, respectively. This macro-averaging approach ensures that each class contributes equally to the evaluation, making the metric robust to class imbalance [115,116].
- Macro-Averaged ROC-AUC: Receiver Operating Characteristic (ROC) curves provide a visual and quantitative tool for evaluating classifier performance across different decision thresholds. Originally developed for medical decision-making, ROC analysis has become widely adopted in machine learning and data mining research. The area under the ROC curve (AUC) offers a single scalar measure of overall classification performance. AUC values range from 0 to 1, where 1 indicates perfect discrimination and 0.5 corresponds to random guessing. In practice, any meaningful classifier should achieve an AUC above 0.5 [117,118].
  For the multi-class problems, we employed a one-vs-rest strategy to compute the ROC curves and AUC values for each class independently [119]. In this approach, each class is treated sequentially as the positive class, while all remaining classes are combined to form the negative class. For our three-class CTG dataset (normal, suspect, pathological), this procedure generated three separate ROC curves. Each curve plots sensitivity (true-positive rate) against 1-specificity (false-positive rate) across all possible classification thresholds. The Macro-Averaged ROC-AUC was then calculated as the arithmetic mean of the individual class AUC values:
  (4) $$\mathrm{Macro}\text{-}\mathrm{AUC}\;=\;\frac{1}{k}\sum_{i=1}^{k}{\mathrm{AUC}}_{i}$$
  where *k* represents the number of classes and $AUC_{i}$ denotes the area under the ROC curve for class *i*. By averaging across classes rather than aggregating predictions, this macro-averaging approach ensures that each class contributes equally to the final metric, regardless of its frequency in the dataset. This property makes the Macro-AUC particularly well-suited for imbalanced classification tasks [120].

We selected these metrics because the CTG dataset is highly imbalanced. Conventional measures such as standard accuracy can obscure poor performance on minority classes. This equal weighting is particularly important in medical applications, where correct classification of rare cases such as pathological fetal conditions is of significant clinical importance. By using these imbalance-aware metrics alongside Balanced Accuracy, we ensured a comprehensive and fair assessment of model performance across all fetal health categories.

## 4. Results and Discussion

**Figure 3:**
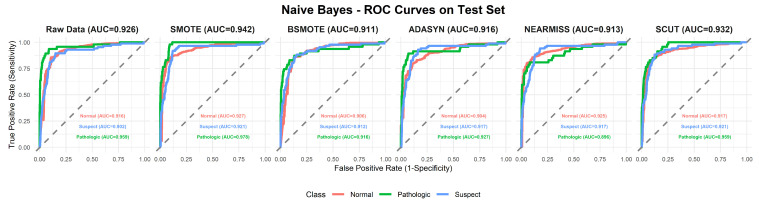
ROC curves for the NB algorithm under resampling methods on the CTG test set (dashed line: AUC = 0.5).

**Figure 4:**
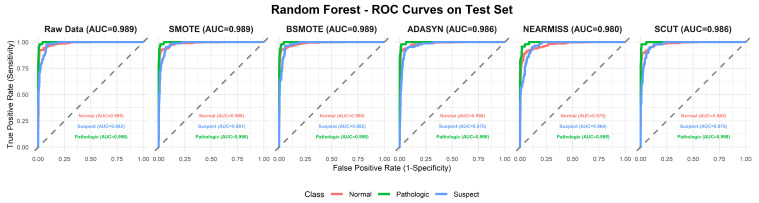
ROC curves for RF algorithm under resampling methods on the CTG test set (dashed line: AUC = 0.5).

**Figure 5:**
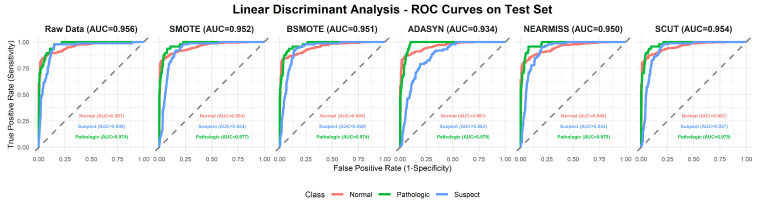
ROC curves for the LDA algorithm under resampling methods on the CTG test set (dashed line: AUC = 0.5).

**Figure 6:**
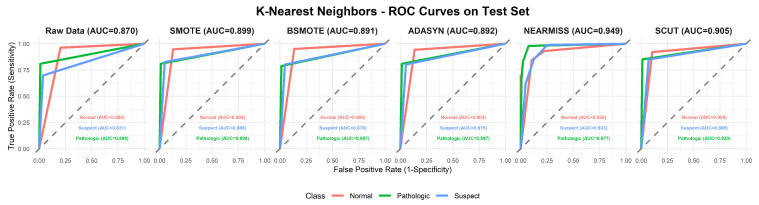
ROC curves for the KNN algorithm under resampling methods on the CTG test set (dashed line: AUC = 0.5).

**Figure 7:**
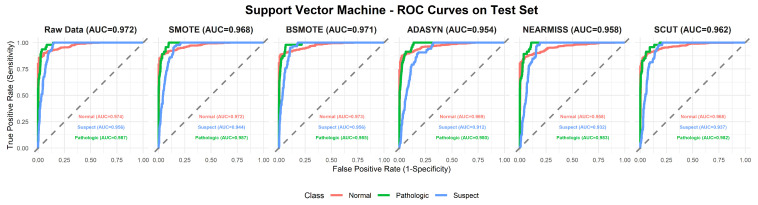
ROC curves for SVM algorithm under resampling methods on the CTG test set (dashed line: AUC = 0.5).

**Figure 8:**
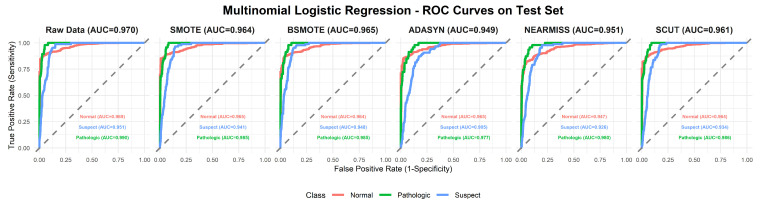
ROC curves for MLR algorithm under resampling methods on the CTG test set (dashed line: AUC = 0.5).

**Figure 9:**
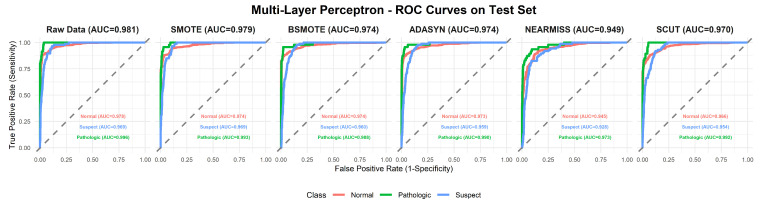
ROC curves for MLP algorithm under resampling methods on the CTG test set (dashed line: AUC = 0.5).

This section provides a comprehensive analysis of the performance of various machine learning models under different resampling methods. The analysis first focuses on overall performance across classifier–resampling combinations (Table 2), then examines class-wise outcomes with clinical relevance (Table 3). The findings are subsequently positioned within the recent CTG literature using Table A1 (Appendix A), followed by an evaluation of performance through ROC analyses (Figure 3, Figure 4, Figure 5, Figure 6, Figure 7, Figure 8 and Figure 9).

Table 2 summarizes the comparative performance of seven machine learning algorithms evaluated under six configurations: the original unbalanced dataset (base) and five resampling methods (SMOTE, BSMOTE, ADASYN, NearMiss, and SCUT). Performance was assessed using four imbalance-aware metrics: BACC, Macro-MCC, Macro-F1, and Macro-Averaged ROC-AUC. The results are analyzed from three complementary perspectives: algorithm-specific performance, resampling method effectiveness, and metric-dependent interpretation.

According to the results in Table 2 the RF classifier consistently emerges as the most reliable model for the CTG dataset across all resampling strategies. The highest BACC (0.9118) is obtained by RF on the base data. Meanwhile, RF combined with BSMOTE yields slightly superior class-balanced performance metrics (Macro-MCC = 0.8533, Macro-F1 = 0.9073) compared with RF-base. This confirms its robustness and stability. The MLP model also performs strongly without resampling (BACC = 0.8636). SMOTE slightly increases its Macro-MCC and Macro-F1 (0.7862 and 0.8564) but lowers BACC. For KNN, the best Macro-MCC and Macro-F1 (0.7820 and 0.8513) are achieved with SMOTE, though the base model retains the highest BACC (0.8473). This indicates that synthetic neighbors improve minority class recall but may slightly blur decision boundaries. For linear or margin-based models (SVM, LDA, NB, MLR), the base datasets generally produce the best BACC values. In contrast, SMOTE or BSMOTE yield modest gains in Macro-MCC and Macro-F1. These trends are consistent with the expected effects of oversampling: shifting class priors and smoothing boundaries.

From a resampling perspective, the base configuration consistently achieved the highest BACC scores across all classifiers. This contrasts with conventional expectations regarding imbalance correction. Among oversampling methods, BSMOTE outperformed SMOTE and ADASYN for ensemble-based models by focusing on borderline minority instances. SMOTE provided moderate Macro-F1 improvements for distance-based and neural network models. ADASYN rarely outperformed alternatives and often yielded the lowest BACC scores due to heavy oversampling in difficult regions. NearMiss’s undersampling consistently degraded performance, underscoring the risk of discarding informative majority class samples. SCUT performed competitively with KNN but did not dominate overall.

From a metric-dependent perspective, evaluation criterion choice significantly influenced model ranking. BACC favored the base configuration, but may overestimate generalization when test data retains original class imbalance. Macro-MCC provided a more conservative estimate, with RF-BSMOTE achieving the highest value. Macro-F1 exhibited similar patterns to BACC but demonstrated greater sensitivity to minority class performance. The strong alignment between Macro-MCC and Macro-F1 trends suggests both metrics reliably capture class-balanced performance. In contrast, BACC may be more susceptible to threshold and prior-shift effects.

Collectively, these results confirm that resampling effectiveness is inherently model dependent. It must be assessed using multiple imbalance-aware metrics rather than relying on overall accuracy alone. RF trained on the base dataset emerged as the optimal model for multi-class fetal health classification from CTG. It balances high BACC with strong class-balanced metrics. BSMOTE offers a competitive alternative for scenarios prioritizing enhanced minority class detection, particularly when paired with ensemble methods. This experimental design serves as an ablation analysis, where comparing each resampling condition against the base (no resampling) baseline within each classifier isolates the specific contribution of resampling to model performance.

To complement these performance metrics, Table 3 presents per-class sensitivity, specificity, and precision values, providing a granular assessment of classifier behavior across fetal health categories. These metrics are particularly relevant for evaluating pathological class detection, where missed cases carry severe clinical consequences. For the normal class, all classifiers achieved high sensitivity (≥0.802), with RF-base reaching 0.974. Specificity values were more variable, ranging from 0.699 (LDA-base) to 0.985 (LDA-ADASYN). Precision for the normal class was consistently high (≥0.923), indicating reliable identification of healthy cases.

For the suspect class, which represents an intermediate clinical category, sensitivity remained challenging across all models. The highest suspect sensitivity was achieved by LDA-SMOTE and LDA-BSMOTE (0.919), while RF-base exhibited lower sensitivity (0.779) despite its overall superior performance. This trade-off reflects the inherent difficulty of distinguishing the suspect class from both normal and pathological categories. Specificity for the suspect class ranged from 0.826 (LDA-ADASYN) to 0.973 (RF-base), indicating variable false-positive rates. Precision values for the suspect class were generally lower than for other classes, with NearMiss and SCUT variants yielding values below 0.52 for several classifiers.

For the pathological class, RF consistently demonstrated the highest sensitivity values, with RF-BSMOTE achieving 0.936 and RF-NearMiss/SCUT reaching 0.957. These results indicate that RF-based models correctly identified over 93% of pathological cases regardless of resampling strategy. In contrast, NB exhibited the lowest pathological sensitivity (0.468 with ADASYN), highlighting its limitations for minority class detection. Regarding specificity, all classifiers maintained values above 0.94 for the pathological class, confirming minimal false-positive rates. Precision for pathological detection was highest with RF-BSMOTE (0.957) and RF-base (0.956), demonstrating that positive predictions for this critical class were highly reliable.

These per-class results complement the aggregate metrics in Table 2 by revealing class-specific trade-offs. Notably, oversampling methods improved pathological sensitivity for most classifiers but occasionally reduced suspect precision due to synthetic sample overlap near class boundaries. The RF classifier maintained the most balanced performance across all three classes, supporting its selection as the preferred model for clinical deployment.

To position our findings within the existing literature, a comparative summary of recent CTG classification studies is provided in Table A1 (Appendix A). The comparison includes study year, classification methods, number of classes, performance metrics, and imbalance handling strategies. Most prior studies reported only overall accuracy, which can be misleading for imbalanced datasets where the majority class dominates performance metrics. While several studies achieved accuracy values exceeding 95%, direct comparison remains difficult due to differences in evaluation metrics, classification schemes, and validation strategies. Among studies employing SMOTE, Ahmed et al. reported a high accuracy (98.9%) and F1-score (99.3%), yet relied primarily on aggregate metrics without per-class evaluation. In contrast, Nazlı et al. reported BACC (91.34%) using SMOTE, which aligns closely with our BACC result for RF (0.9118). This consistency across independent studies using balanced metrics strengthens confidence in the reported performance levels for three-class CTG classification.

To complement Table 2, Figure 3, Figure 4, Figure 5, Figure 6, Figure 7, Figure 8 and Figure 9 illustrate the ROC curves for each classifier under all resampling conditions. Each panel presents the macro-averaged AUC along with class-specific curves for the normal, suspect, and pathologic categories. This provides a comprehensive view of model discrimination performance across different resampling strategies. The algorithm-specific findings are summarized as follows:

1. NB (Figure 3): SMOTE achieves the highest performance (AUC = 0.942) compared to the base model (0.926), whereas BSMOTE and NearMiss reduce it to approximately 0.911–0.913. Despite these AUC improvements, BACC decreases under both SMOTE (0.8128 to 0.7671) and SCUT (0.8128 to 0.7013), demonstrating that enhanced ranking ability does not guarantee improved threshold-based classification accuracy.
2. RF (Figure 4): This achieves near-perfect AUCs (approximately 0.986–0.989) across all resampling methods, confirming its robustness and superior predictive calibration. The base, SMOTE, and BSMOTE models achieve identical top performance (AUC = 0.989), indicating highly stable and accurate class separation. Minor declines under ADASYN, NearMiss, and SCUT suggest that resampling offers no additional benefit.
3. LDA (Figure 5): This maintains high AUCs across methods (approximately 0.951–0.956), with a minor reduction under ADASYN (0.934). The base configuration remains optimal.
4. KNN (Figure 6): Overall AUC improves from the base model (0.870) under SMOTE (0.899) and SCUT (0.905), while NearMiss achieves the highest value (0.949). However, BACC consistently decreases across all configurations despite these AUC gains. This divergence occurs because improving ranking ability (AUC) does not guarantee better performance at a fixed decision threshold; resampling shifts class priors and alters optimal threshold positions, resulting in improved discrimination without corresponding gains in classification accuracy.
5. SVM (Figure 7): This records excellent performance with base and BSMOTE models (approximately 0.971–0.972), while ADASYN and NearMiss slightly lower AUCs to approximately 0.954–0.958.
6. MLR (Figure 8): This shows consistently strong performance (AUC approximately 0.964–0.970), with the base model performing best (0.970).
7. MLP (Figure 9): The base model exhibits the highest AUC (0.981), while oversampling slightly decreases performance (SMOTE = 0.979, BSMOTE and ADASYN= 0.974).

## 5. Conclusions

This study evaluated seven machine learning algorithms across six resampling configurations for multi-class fetal health classification from CTG data under severe class imbalance. Using four imbalance-robust metrics (BACC, Macro-MCC, Macro-F1, and Macro-AUC), we demonstrated that RF consistently emerges as the most reliable classifier. RF trained on the original unbalanced dataset achieved the highest BACC (0.9118), while RF combined with BSMOTE delivered superior class-balanced performance (Macro-MCC = 0.8533, Macro-F1 = 0.9073) with near-perfect AUC values (0.986–0.989). MLP also demonstrated strong performance without resampling, whereas linear and probabilistic models generally performed best on the base configuration. These patterns remained consistent across both threshold-based metrics (Table 2) and ROC curve analyses (Figure 3, Figure 4, Figure 5, Figure 6, Figure 7, Figure 8 and Figure 9).

Resampling effects proved to be model-dependent, with notable differences in how algorithms responded to class balance manipulation. While RF, MLP, and linear models achieved optimal BACC on the natural class distribution, oversampling techniques, particularly SMOTE and BSMOTE, demonstrated significant improvements in minority class discrimination and class-balanced metrics across multiple model families. Specifically, KNN benefited substantially from SMOTE, exhibiting enhanced Macro-F1 (from 0.8346 to 0.8513) and Macro-MCC (from 0.7574 to 0.7820) without sacrificing overall accuracy. Similarly, MLP with SMOTE showed improved class-balanced metrics (Macro-MCC = 0.7862, Macro-F1 = 0.8564) and enhanced minority class recall. These findings demonstrate that strategic oversampling can translate improved class discrimination into clinically meaningful performance gains for specific model architectures, particularly distance-based and neural network methods.

The superiority of the base configuration for BACC can be explained by three factors: First, synthetic oversampling may introduce noise or unrealistic feature combinations that do not reflect the true underlying data distribution. Second, the test set retains the original class imbalance (normal: 77.8%, suspect: 13.9%, pathological: 8.3%), creating a distributional mismatch with oversampled training data that affects generalization. Third, models trained on balanced data may optimize decision boundaries that are suboptimal for the naturally imbalanced distribution encountered in clinical practice. Despite this, BSMOTE consistently improved class-balanced metrics across ensemble methods, confirming its value for applications where enhanced minority class detection is critical.

Importantly, improvements in AUC did not always lead to higher BACC; certain model–resampling combinations exhibited improved AUC despite decreased BACC, highlighting the distinction between ranking-based and threshold-based performance metrics. This pattern was observed in NB with SMOTE and SCUT as well as KNN, across all resampling methods. This divergence confirms that resampling can enhance ranking ability while reducing threshold-based accuracy due to class prior shifting effects.

Per-class results further highlighted clinically relevant differences. For the pathological class, RF achieved the highest sensitivity (0.957 with SCUT) and precision (0.957 with BSMOTE), ensuring reliable detection of high-risk cases. The suspect class remained the most challenging category, with lower sensitivity values across all classifiers due to its intermediate clinical characteristics. The normal class exhibited consistently high sensitivity and precision across all models. These findings emphasize that aggregate metrics alone may obscure clinically important class-specific variations, reinforcing the need for per-class evaluation in imbalanced medical classification tasks.

From a clinical perspective, these findings suggest that RF trained on the original class distribution offers the most reliable approach for routine fetal health monitoring, where pathological cases naturally occur at low frequency. The model’s consistently high performance across all metrics, combined with its robustness to resampling variations, makes it particularly suitable for deployment in clinical decision support systems. For scenarios prioritizing enhanced detection of rare pathological cases, such as high-risk obstetric units or screening programs, RF combined with BSMOTE, or alternatively KNN with SMOTE, provides compelling options that balance overall accuracy with improved minority class sensitivity. Methodologically, this study demonstrates that evaluating classifier performance under class imbalance requires multiple complementary metrics rather than relying on overall accuracy alone. The choice of resampling method must be tailored to the specific algorithm architecture and clinical priorities.

While these results are promising for clinical decision support, several limitations warrant consideration: First, this study utilized a single publicly available dataset from one institution, which may limit generalizability to different clinical settings, populations, or CTG acquisition devices. However, the UCI CTG dataset is widely used in fetal health research, allowing comparison with existing studies. Furthermore, testing five resampling methods across seven classifiers provides methodological guidance applicable to other imbalanced datasets. Nevertheless, the optimal resampling strategy may not directly transfer, and external validation is recommended before clinical deployment. Second, this study relies on preprocessed CTG features rather than raw signal traces, which may discard temporal patterns beneficial for classification. Moreover, the dataset lacks clinical variables such as maternal age, gestational age, and medical history, limiting comprehensive clinical modeling. Third, while we employed stratified train–test splitting and cross-validation to prevent data leakage, the relatively small sample size of minority classes (295 suspect, 176 pathologic) may have affected the stability of performance estimates. Additionally, the specific impact of each methodological element cannot be fully quantified in isolation. As a benchmarking study, our design compares model–resampling combinations; decomposing individual contributions would require factorial experimental designs.

Future research should extend this work in several directions: First, these methods should be evaluated on additional CTG datasets from different institutions to assess generalizability across diverse clinical populations and data distributions. Second, deep learning methods could be applied to raw CTG signals integrated with clinical metadata to capture temporal patterns and enable comprehensive clinical modeling. Third, data augmentation techniques or larger datasets with more balanced class distributions could improve the stability of performance estimates for minority classes. Fourth, future studies should employ repeated stratified cross-validation to report mean ± standard deviation and apply statistical significance tests (e.g., Friedman test, McNemar’s test) for more robust performance comparisons. Fifth, additional machine learning methods such as advanced ensemble algorithms (XGBoost, CatBoost, LightGBM) and deep learning architectures could be evaluated to determine whether they outperform the classical methods tested here. Sixth, other resampling techniques such as SMOTE variants (Borderline-SMOTE2, SVM-SMOTE) and hybrid methods combining oversampling and undersampling warrant investigation. Seventh, detailed misclassification analysis should be conducted to identify feature patterns associated with incorrectly classified pathological cases, providing valuable insights for clinical decision-making. Eighth, feature importance analysis and model explanation techniques (e.g., SHAP, permutation importance) should be incorporated to identify which CTG attributes most strongly influence classification decisions. Finally, additional imbalance-aware metrics such as G-mean, Cohen’s Kappa, and class-specific precision–recall curves could provide complementary perspectives on classifier performance.

## Figures and Tables

**Table 1:** Description of cardiotocogram (CTG) attributes used in the model.

Symbol	Variable Description
Class	Fetal state class: normal; suspect; pathologic
LB	Fetal heart beats per minute
AC	Accelerations per second
FM	Fetal movements per second
UC	Number of uterine contractions per second
DL	Number of light decelerations per second
DS	Number of severe decelerations per second
DP	Number of prolonged decelerations per second
ASTV	Percentage of time with abnormal short-term variability
MSTV	Mean value of short-term variability
ALTV	Percentage of time with abnormal long-term variability
MLTV	Mean value of long-term variability
Width	Width of FHR histogram
Max	Maximum of FHR histogram
Min	Minimum of FHR histogram
Nmax	Number of histogram peaks
Nzeros	Number of histogram zeros
Mode	Histogram mode
Mean	Histogram mean
Median	Histogram median
Variance	Histogram variance
Tendency	Histogram tendency: −1 = left asymmetric; 0 = symmetric; 1 = right asymmetric

**Table 2:** Comparison of classifier performance across resampling techniques on the CTG dataset.

Algorithm	Resampling Method	BACC	Macro-MCC	Macro-F1
NB	base	0.8128	0.6474	0.7550
	SMOTE	0.7671	0.6511	0.7616
	BSMOTE	0.7914	0.6340	0.7474
	ADASYN	0.7787	0.6061	0.7256
	NearMiss	0.7083	0.6210	0.7288
	SCUT	0.7013	0.6182	0.7276
RF	base	0.9118	0.8477	0.9003
	SMOTE	0.8897	0.8428	0.8973
	BSMOTE	0.9092	0.8533	0.9073
	ADASYN	0.8815	0.8437	0.8957
	NearMiss	0.7880	0.7616	0.8357
	SCUT	0.8657	0.8283	0.8898
LDA	base	0.7874	0.6540	0.7626
	SMOTE	0.7041	0.6415	0.7352
	BSMOTE	0.7209	0.6574	0.7490
	ADASYN	0.6490	0.5880	0.6916
	NearMiss	0.7005	0.6315	0.7324
	SCUT	0.7018	0.6359	0.7313
KNN	base	0.8473	0.7574	0.8346
	SMOTE	0.8449	0.7820	0.8513
	BSMOTE	0.8375	0.7713	0.8413
	ADASYN	0.8312	0.7658	0.8401
	NearMiss	0.7479	0.6820	0.7850
	SCUT	0.8245	0.7679	0.8445
SVM	base	0.8190	0.6946	0.7835
	SMOTE	0.7575	0.7042	0.7855
	BSMOTE	0.7791	0.7150	0.7932
	ADASYN	0.7013	0.6556	0.7429
	NearMiss	0.7193	0.6654	0.7566
	SCUT	0.7546	0.6903	0.7793
MLR	base	0.7742	0.6683	0.7699
	SMOTE	0.7437	0.6924	0.7770
	BSMOTE	0.7533	0.6908	0.7801
	ADASYN	0.6914	0.6393	0.7340
	NearMiss	0.7073	0.6355	0.7443
	SCUT	0.7382	0.6753	0.7649
MLP	base	0.8636	0.7849	0.8560
	SMOTE	0.8326	0.7862	0.8564
	BSMOTE	0.8230	0.7610	0.8391
	ADASYN	0.8040	0.7509	0.8306
	NearMiss	0.7527	0.6779	0.7797
	SCUT	0.7852	0.7219	0.8126

Note: A detailed comparison with existing CTG classification studies is provided in Table A1 (Appendix A).

**Table 3:** Per-class sensitivity (recall), specificity, and precision (PPV) across classifiers and resampling strategies on the CTG dataset.

Algorithm	Resampling Method	Sensitivity/Recall			Specificity			Precision/PPV		
		Normal	Suspect	Pathological	Normal	Suspect	Pathological	Normal	Suspect	Pathological
NB	base	0.935	0.674	0.575	0.722	0.924	0.997	0.927	0.580	0.931
	SMOTE	0.901	0.779	0.638	0.797	0.908	0.987	0.944	0.568	0.790
	BSMOTE	0.921	0.721	0.553	0.729	0.919	0.993	0.928	0.579	0.867
	ADASYN	0.897	0.802	0.468	0.722	0.904	0.993	0.925	0.566	0.846
	NearMiss	0.834	0.884	0.660	0.902	0.851	0.975	0.970	0.481	0.674
	SCUT	0.828	0.884	0.681	0.902	0.851	0.971	0.970	0.481	0.653
RF	base	0.974	0.779	0.915	0.857	0.973	0.997	0.963	0.817	0.956
	SMOTE	0.956	0.849	0.915	0.902	0.958	0.995	0.974	0.760	0.935
	BSMOTE	0.966	0.814	0.936	0.872	0.969	0.997	0.966	0.805	0.957
	ADASYN	0.953	0.872	0.915	0.925	0.955	0.993	0.980	0.750	0.915
	NearMiss	0.903	0.849	0.957	0.932	0.931	0.971	0.981	0.658	0.726
	SCUT	0.929	0.884	0.957	0.932	0.935	0.995	0.981	0.679	0.938
LDA	base	0.953	0.570	0.702	0.699	0.953	0.985	0.923	0.653	0.786
	SMOTE	0.812	0.919	0.745	0.977	0.832	0.970	0.993	0.459	0.660
	BSMOTE	0.844	0.919	0.702	0.955	0.859	0.973	0.986	0.503	0.674
	ADASYN	0.802	0.733	0.851	0.985	0.826	0.946	0.995	0.396	0.556
	NearMiss	0.814	0.872	0.766	0.955	0.833	0.970	0.986	0.449	0.667
	SCUT	0.822	0.895	0.723	0.970	0.837	0.970	0.991	0.461	0.654
KNN	base	0.962	0.698	0.809	0.797	0.964	0.988	0.947	0.750	0.844
	SMOTE	0.947	0.826	0.809	0.872	0.951	0.988	0.966	0.725	0.844
	BSMOTE	0.951	0.802	0.787	0.865	0.953	0.987	0.964	0.726	0.822
	ADASYN	0.943	0.802	0.809	0.865	0.948	0.987	0.964	0.704	0.826
	NearMiss	0.846	0.872	0.851	0.895	0.875	0.978	0.968	0.521	0.755
	SCUT	0.921	0.849	0.851	0.895	0.929	0.988	0.971	0.652	0.851
SVM	base	0.951	0.698	0.638	0.782	0.937	0.993	0.943	0.632	0.882
	SMOTE	0.879	0.861	0.787	0.962	0.880	0.980	0.989	0.529	0.755
	BSMOTE	0.899	0.872	0.723	0.940	0.895	0.985	0.983	0.564	0.791
	ADASYN	0.875	0.756	0.809	0.970	0.882	0.959	0.991	0.500	0.613
	NearMiss	0.846	0.872	0.787	0.962	0.862	0.970	0.988	0.497	0.673
	SCUT	0.873	0.837	0.787	0.940	0.875	0.981	0.982	0.511	0.771
MLR	base	0.937	0.663	0.702	0.782	0.935	0.983	0.942	0.613	0.767
	SMOTE	0.877	0.849	0.787	0.955	0.884	0.975	0.987	0.533	0.712
	BSMOTE	0.893	0.837	0.745	0.910	0.902	0.976	0.974	0.571	0.714
	ADASYN	0.867	0.744	0.809	0.947	0.882	0.956	0.984	0.496	0.594
	NearMiss	0.848	0.802	0.787	0.895	0.871	0.968	0.968	0.493	0.661
	SCUT	0.875	0.837	0.745	0.940	0.879	0.976	0.982	0.518	0.714
MLP	base	0.962	0.733	0.851	0.820	0.966	0.990	0.953	0.768	0.870
	SMOTE	0.935	0.826	0.894	0.895	0.946	0.985	0.971	0.703	0.824
	BSMOTE	0.929	0.802	0.851	0.880	0.933	0.988	0.967	0.651	0.851
	ADASYN	0.915	0.814	0.872	0.902	0.922	0.985	0.973	0.620	0.820
	NearMiss	0.873	0.826	0.787	0.880	0.890	0.980	0.965	0.538	0.755
	SCUT	0.907	0.791	0.851	0.872	0.920	0.981	0.964	0.607	0.784

## Data Availability

The original data presented in the study is openly available in the UCI ML Repository at https://archive.ics.uci.edu/dataset/193/cardiotocography (accessed on 20 September 2024).

## Appendix A

Detailed performance comparisons of the proposed method with existing CTG classification studies are presented in Table A1.

**Table A1:** Performance comparison of the proposed method with existing CTG classification studies.

Study	Year	Methods	Classes	Performance	Imbalance Handling
Sahin et al. [25]	2015	ANN, SVM, k-NN, RF, CART, LR, C4.5, RBFN	Binary	Accuracy = 99.2%; F1-score = 99.2%; AUC = 99.9%	No
Piri et al. [29]	2019	DT, LR, SVM, KNN, GNB, RF, XGBoost	3-class	Accuracy = 84%	No
Piri et al. [31]	2020	MOGA-CD + DT, SVM, GNB, RF, XGBoost	3-class	Accuracy = 94%	No
Pradhan et al. [34]	2021	RF, LR, KNN, GBM	3-class	Accuracy = 93%	No
Rahmayanti et al. [35]	2022	ANN, LSTM, XGBoost, SVM, KNN, LightGBM, RF	3-class	Accuracy = 89–99%; F1-score = 98%; AUC = 99%	SMOTE
Kaliappan et al. [36]	2023	DT, RF, SVM, KNN, GNB, AdaBoost, Gradient Boosting, Voting Classifier, Neural Networks	3-class	Accuracy = 99%; F1-score ≈ 97%; MCC = 96%	Random Oversampling
Salini et al. [38]	2024	RF, LR, DT, SVC, KNN, Voting Classifier	3-class	Accuracy = 93%	No
Nazlı et al. [40]	2025	CatBoost, DT, ExtraTrees, GB, KNN, LightGBM, RF, SVM, ANN, DNN	3-class	BACC = 91.34%	SMOTE
Ahmed et al. [13]	2025	ML (RF, SVM, KNN, DT, LR, XGBoost, ET); DL (ANN, CNN, RNN, LSTM, GRU) + Stacking Meta-Classifier	3-class	Accuracy = 98.9%; F1-score = 99.3%; AUC = 99.8%	SMOTE
Bhukya et al. [41]	2025	ML: KNN, DT, SVM, RF, SGD, GB, AdaBoost, XGBoost; DL: CNN, LSTM, BiLSTM	3-class	Accuracy ≈ 99.39%; F1-score = 99%	No
Proposed work	2026	NB, RF, LDA, KNN, Linear SVM, MLR, MLP	3-class	BACC = 91.18% (RF + Base), 90.92% (RF + BSMOTE); Macro-F1 = 90.73%; Macro-MCC = 85.33%; Macro-ROC-AUC = 98.9% (RF + BSMOTE)	Base, SMOTE, BSMOTE, ADASYN, NearMiss, SCUT
